# Impact of Pluronic F-127 on the Stability of Quercetin-Loaded Liposomes: Insights from DSC Preformulation Studies

**DOI:** 10.3390/ma17225454

**Published:** 2024-11-08

**Authors:** Effrosyni-Maria Kosti, Heliana Sotiropoulou, Ioannis Tsichlis, Maria Tsakiri, Nikolaos Naziris, Costas Demetzos

**Affiliations:** Section of Pharmaceutical Technology, Department of Pharmacy, School of Health Sciences, National and Kapodistrian University of Athens, Panepistimioupolis Zografou, 15771 Athens, Greece; efrosinikosm@hotmail.com (E.-M.K.); helian22so@gmail.com (H.S.); gtsichlis@pharm.uoa.gr (I.T.); tsakirim@pharm.uoa.gr (M.T.);

**Keywords:** liposomes, drug delivery nanosystems, Pluronic F-127, quercetin, stability, DSC

## Abstract

The aim of the present study is to evaluate the stability of DMPC:Pluronic F-127 and DPPC:Pluronic F-127 liposomes, both with and without incorporated quercetin. Quercetin belongs to the class of flavonoids and has shown antioxidant, antiviral, anti-inflammatory, anti-cancer, and antimicrobial activities. Dynamic light scattering, electrophoretic light scattering, and differential scanning calorimetry (DSC) were utilized to investigate the cooperative behavior between liposomal components and its effect on stability. All formulations were stored at 4 °C and 25 °C and studied over 42 days. Furthermore, the interaction of the final formulations with serum proteins was assessed to evaluate the potential of Pluronic F-127 as a stabilizer in these liposomal nanosystems. This study highlights the impact of DSC in preformulation evaluations by correlating thermal behavior with quercetin incorporation and variations in size and the polydispersity index. According to the results, quercetin increased the fluidity and stability of liposomal nanosystems, while Pluronic F-127 was not sufficient for effective steric stabilization. Additionally, DSC thermograms revealed the integration of Pluronic F-127 into lipid membranes and showed phase separation in the DMPC nanosystem. In conclusion, the results indicate that the DPPC:Pluronic F-127:quercetin nanosystem exhibited the desired physicochemical and thermotropic properties for the effective delivery of quercetin for pharmaceutical purposes.

## 1. Introduction

Liposomes were first discovered in the 1960s by Dr. Alec D. Bangham and his team [1]. Liposomes are defined as colloidal, nearly spherical vesicles consisting of single or multiple lipid bilayers encapsulating an internal aqueous core [2,3,4]. The lipid bilayers are formed due to the ability of amphiphilic lipid molecules, such as phospholipids, to self-assemble in aqueous environment [3]. Due to their unique structure, liposomes can entrap lipophilic and hydrophilic molecules into the lipid bilayer and in the aqueous core, respectively, as well as amphiphilic molecules at the water/lipid bilayer interface [2,3,5,6]. Liposomes have been studied extensively in pharmaceutical research as drug delivery systems due to their biocompatibility, biodegradability, and non-toxic nature [2,3,7]. Moreover, the similarity of liposomes’ membrane to plasma membrane facilitates their interaction with human cells and enhances the cellular uptake of bioactive molecules [3]. Additional advantages of liposomes include the protection of the incorporated drug from chemical and/or biological degradation, tissue-specific drug delivery, and the decrease in systemic adverse effects. Moreover, modification of the liposome surface with ligands can allow the active targeting of cells [3,6]. All the above properties make liposomes suitable carriers for a wide range of biomedical applications [2,5,8].

Despite the multiple advantages that liposomes offer as drug delivery systems, their application has certain limitations, including low entrapment efficiency, biological instability, and a complex pharmacokinetic and pharmacodynamic profile [3]. Several factors can influence the biodistribution and efficacy of liposomes loaded with bioactive molecules, including liposome composition, size, surface modifications, and interactions with biological systems. Therefore, to address these challenges and improve their clinical efficacy, several strategies are adopted, such as surface functionalization, the incorporation of stimuli-responsive biomaterials, and the optimization of the lipid composition. Regarding biological instability, the interaction of liposomes with blood proteins leads to an activation of the reticuloendothelial system (RES), resulting in a short blood circulation time [3,5]. Proteins in the bloodstream can bind to the surface of conventional liposomes and form the so-called protein corona, which mainly consists of opsonins. The protein corona is recognized by the phagocytes of the mononuclear phagocyte system (MPS), leading to liposome elimination [3,5,9]. Thus, the need to reduce the immunogenicity of liposomes becomes apparent and can be achieved by coating their surface with biocompatible hydrophilic polymers [3]. The polymer mainly used for long-circulating (stealth) liposomes is polyethylene glycol (PEG). As the flexible polymer chains create a hydrophilic coating around liposomes, PEGylation can prolong the blood half-life of liposomes through the development of repulsive forces between the hydrophilic surfaces of liposomes and opsonins due to steric and enthalpic stabilization [10,11,12,13]. The above results in delaying or hindering the onset of the opsonization process and therefore increasing the retention of liposomes at the target tissue, which is a key factor for their therapeutic effects [11,12].

PEGylation is one of the most common approaches for increasing blood circulation time and improving drug efficacy. However, several limitations have been reported throughout the years, potentially limiting its applications [12,14]. First of all, the use of PEG may cause leakage of the encapsulated drug [15]. Moreover, the non-biodegradable nature of PEG can trigger an immune response, leading to the production of anti-PEG antibodies, which have been associated with hypersensitivity reactions, although the underlying mechanisms have not been fully clarified [14,16]. Thus, current research is focused on alternative methods to create nanoparticles with prolonged circulation time [12]. Apart from PEG, an alternative that is widely used for the stabilization of liposomes due to its commercial availability and low cost are block copolymers, such as Pluronic F-127 [17,18]. Pluronic F-127 (also known as Poloxamer 407) is a triblock nonionic copolymer consisting of two hydrophilic polyethylene oxide (PEO) blocks at the ends of its structure, enclosing a central hydrophobic polypropylene oxide (PPO) block [19,20]. The hydrophilic blocks of the copolymer extend into the external aqueous environment of the liposomes [19] and prevent their fusion, as well as their interaction with blood proteins, both due to steric and enthalpic repulsion forces that develop during the interaction of their hydrophilic surfaces [13,17,21,22]. Additional advantages of the incorporation of Pluronic F-127 in the structure of liposomes compared to PEG include reduced particle size [20,21], enhanced drug bioavailability [21,22], and biodegradability [18].

Besides the above advantages, it is crucial to examine the unique characteristics that Pluronic F-127 exhibits as a novel biomaterial. Due to the complex and thermoresponsive behavior of Pluronic F-127, it is essential to study the effect of temperature using a thermal analysis technique, such as DSC, on novel systems containing Pluronic F-127. DSC is a highly sensitive technique that provides extensive information about the system under study, including its thermodynamic properties, by detecting thermally induced phase transitions. Since the thermotropic behavior of lipid bilayers has been extensively studied [23,24,25], it is also necessary to understand in depth the interaction of lipids with novel polymers, such as Pluronic F-127. The effect of Pluronic F-127 on the thermodynamic profile of the lipid membrane is of great interest and could greatly contribute to the preformulation studies of drug delivery systems.

Flavonols, a subcategory of polyphenols, exhibit strong antioxidant activity. Quercetin is a typical example of flavonol. Quercetin offers numerous benefits to human health, not only due to its antioxidant activity but also due to its antiviral, anti-inflammatory, anti-cancer, and antimicrobial activity. Recently, “the paradox of quercetin” has been proposed, suggesting that, while quercetin offers protective effects, it can also be converted into a potentially toxic product [26]. Despite the above benefits, quercetin delivery is challenging due to its low solubility and poor oral absorption [27]. To overcome these obstacles, new nanoformulations have been developed to enhance the delivery of hydrophobic molecules, such as quercetin [28,29].

This study aims to develop chimeric liposomal nanosystems and evaluate their physicochemical characteristics and thermotropic behavior. Therefore, we prepared empty and quercetin-loaded DPPC (1,2-dipalmitoyl-sn-glycero-3-phosphocholine) and DMPC (1,2-dimyristoyl-sn-glycero-3-phosphocholine) liposomes, using Pluronic F-127 as an alternative to PEG for physical stabilization. Initially, the physicochemical characteristics of the prepared liposomal nanosystems were studied over a period of 42 days. Dynamic and electrophoretic light scattering were used to determine the hydrodynamic diameter (D_h_), the polydispersity index (PDI), and the zeta potential of the nanosystems. DSC was utilized to study the thermotropic behavior of lipid bilayers over a range of temperatures and after repeated heating–cooling cycles, demonstrating its potential predictive value for preformulation liposomal stability studies. Finally, the interactions between liposomal components and their effect on system stability were investigated, along with the ability of Pluronic F-127 to act as a stabilizer for liposomes against serum proteins.

## 2. Materials and Methods

### 2.1. Materials

**Figure 1:**
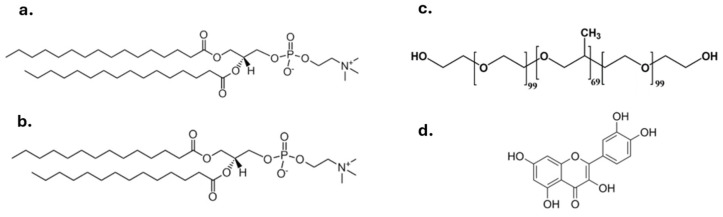
The chemical structures of (**a**) DPPC, (**b**) DMPC, (**c**) Pluronic F-127, and (**d**) quercetin.

The lipids used for the preparation of the lipid-based nanosystems were 1,2-dihexadecanoyl-sn-glycero-3-phosphocholine (DPPC) and 1,2-ditetradecanoyl-sn-glycero-3-phosphocholine (DMPC), which were purchased from Avanti Polar Lipids Inc., (Alabaster, AL, USA) and used without further purification (Figure 1). The nonionic surfactant, Pluronic^®^ F-127 (Poloxamer P407) with a structure of PEO_98_-PPO_67_-PEO_98_ and an average molar mass of 12,600 g/mol, was obtained from Sigma-Aldrich Chemical Co. (St. Louis, MO, USA). Quercetin was purchased from Fluka, BioChemika (Buchs, Switzerland). A Fetal Bovine Serum (FBS) was obtained from Fischer Scientific (Oxford, UK), while chloroform, methanol, and H2O used were of HPLC grade and purchased from Fisher Scientific (UK).

### 2.2. Methods

#### 2.2.1. Preparation of Liposomal Nanosystems

In this study, four liposomal nanosystems were prepared by the thin-film hydration method. The empty liposomes consisted of DPPC:Pluronic F-127 (7:0.17 molar ratio), and DMPC:Pluronic F-127 (7:0.17 molar ratio). These nanosystems were utilized to incorporate quercetin in an initial formulated concentration of 0.25 mg/mL. Specifically, these nanosystems included DPPC:Pluronic F-127:quercetin (7:0.17:0.5 molar ratio) and DMPC:Pluronic F-127:quercetin (7:0.17:0.5 molar ratio). Appropriate amounts of DPPC or DMPC were mixed with the nonionic surfactant (Pluronic^®^ F-127), dissolved in chloroform, and transferred into a round-bottom flask. Using a rotary evaporator (Rota vapor R-114, Buchi, Switzerland), the vacuum was applied, and the solvent was evaporated at 40 °C for 30 min to form a thin film on the flask wall. The developed film was then hydrated with HPLC-grade water to achieve a final lipid concentration of 10 mg/mL by gentle stirring for 1 h in a water bath above the phase transition temperatures of DPPC (41 °C) and DMPC (24 °C), respectively. The resulting multilamellar vesicles (MLVs) were subjected to two sonication cycles (amplitude 70, cycle 0.7) of 5 min each, with a 5 min rest between cycles, using a probe sonicator (UP 200S, dr. Hielsher GmbH, Berlin, Germany). The resulting small unilamellar vesicles (SUVs) were then allowed to anneal for 30 minutes [28,30]. Regarding the quercetin-loaded liposomes, quercetin was dissolved with the other components in a mixture of ethanol/chloroform (1:3 *v*/*v*). Finally, for the physicochemical studies, the DPPC:Pluronic F-127:quercetin liposomal nanosystem was filtered through a 0.45 um syringe filter to remove potential quercetin precipitates [31].

#### 2.2.2. Dynamic and Electrophoretic Light Scattering

The physicochemical characteristics of the nanosystems were assessed by measuring the mean hydrodynamic diameter (D_h_), polydispersity index (PDI), and ζ-potential. The mean hydrodynamic diameter and the polydispersity index (PDI) were obtained by dynamic light scattering (DLS), while the ζ-potential was measured by electrophoretic light scattering (ELS). Each sample (50 μL) was diluted with 1000 μL of HPLC-grade water. Measurements were performed at a detection angle of 90 °C, at 25 °C, in a photon correlation spectrometer (Zetasizer ZSU3105, Malvern, UK), with data analyzed using the ZS XPLORER software 3.3.042 (Malvern software). Measurements were performed in triplicate, and standard deviation was calculated and shown in the graphs.

#### 2.2.3. Incubation in Fetal Bovine Serum

Following their preparation, liposomes were dispersed in an FBS (t = 0 d). These studies were performed by diluting 50 μL of liposomal dispersion with 1000 μL of FBS. Finally, nanosystems were incubated at room temperature for 15 min and light scattering measurements were conducted in the biological medium [32,33].

#### 2.2.4. Entrapment Efficiency Determination

Free quercetin was separated from quercetin entrapped in nanosystems using the ultrafiltration centrifugal method. Specifically, the dispersion was centrifuged for 45 min at 8000 rpm using centrifugal filter tubes [molecular weight (MW) cutoff = 10 kDa; Millipore] at 4 °C. The nanoparticles were separated from the aqueous phase, while free quercetin remained in the supernatant and analyzed using the UV–Vis spectrophotometry method. The free drug concentration was measured at 372 nm with a UV–Vis spectrophotometer (Shimadzu PharmaSpec UV-1700 UV–Vis spectrophotometer, Meckenheim, Germany) using a pre-constructed calibration curve. Centrifuged samples of the respective empty nanosystems were used as a blank. The Entrapment Efficiency (EE)% was calculated using the following equation:

$$(\mathrm{EE})\%\;=\;(1\;-\;\frac{C_{supernatant}}{C_{total}})\%$$

where *C_supernatant_* is the quercetin concentration that was quantified in the supernatant (non-entrapped), and *C_total_* is the total concentration of the quercetin added in the dispersion [28].

#### 2.2.5. Differential Scanning Calorimetry

Differential Scanning Calorimetry experiments were conducted using a DSC822e (Mettler Toledo, Schwerzenbach, Switzerland) calorimeter, calibrated with pure indium (Tm = 156.6 °C). Samples were placed in 40 μL sealed aluminum crucibles. Lipid bilayers consisting of DPPC, DMPC, DPPC:Pluronic F-127 (7:0.17 molar ratio), DMPC:Pluronic F-127 (7:0.17 molar ratio), DPPC:Pluronic F-127:quercetin (7:0.17 molar ratio) and DMPC:Pluronic F-127:quercetin (7:0.17 molar ratio) were prepared by mixing the appropriate aliquots of the stock solutions into a round-bottom flask. The solvents were evaporated under vacuum at 40 °C for 30 min using a rotary evaporator (Rota vapor R-114, Buchi, Switzerland) to form a thin film. The film was stored under vacuum in a desiccator for at least 24 h to eliminate residual solvent. The prepared lipid bilayers were analyzed by placing approximately 4 mg of each sample in a crucible and hydrating with 50% *w*/*v* of HPLC-grade water. For all the experiments, one heating–cooling cycle was conducted, followed by additional heating to assess the thermodynamic properties of the systems.

#### 2.2.6. Statistical Analysis

Measurements were performed in triplicate, and standard deviation (SD) was calculated and shown in the graphs. A statistical analysis was performed on the size and the polydispersity index by utilizing a two-tailed paired Student’s *t*-test for protein interaction studies and a one-way ANOVA followed by a Bonferroni post hoc test for physical stability studies. *p*-values < 0.05 were considered statistically significant. The statistical analysis was performed by using a GraphPad Prism 8.0 software package. The significance of comparisons is presented on the graphs.

## 3. Results

### 3.1. Physicochemical Characterization of the Non-Loaded Nanoparticles

Regarding the DPPC:Pluronic F-127 and DMPC:Pluronic F-127 nanosystems, physicochemical characterization was carried out for a period of 42 days at two different storage temperatures (Table 1).

**Figure 2:**
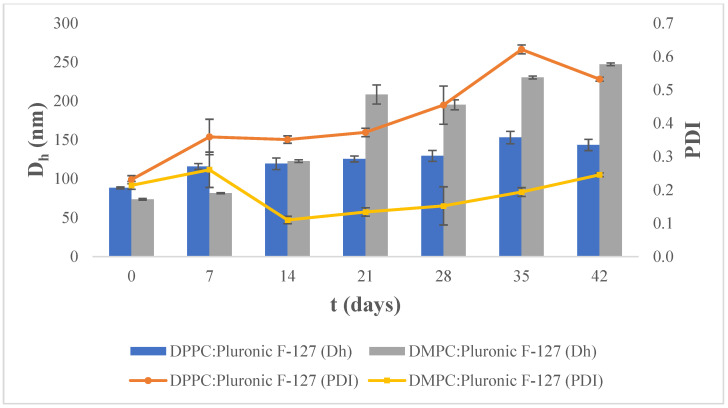
Stability assessment of the size (D_h_, nm) and polydispersity index (PDI) of DPPC:Pluronic F-127 and DMPC:Pluronic F-127 7:0.17 molar ratio over time at 4 °C. For D_h_ of all nanosystems, *p*-value < 0.0001, and for PDI, *p*-value < 0.0001.

At 4 °C, the mean hydrodynamic diameter and polydispersity index of the DPPC:Pluronic F-127 system were increased gradually over time (Figure 2). An increase in the polydispersity index indicates the presence of multiple size populations within the colloidal dispersion.

For the DMPC:Pluronic F-127 nanosystem stored at 4 °C, the mean hydrodynamic diameter (D_h_) gradually increased over 42 days, leading to the collapse of the nanosystem. On the other hand, at 42 days, the polydispersity index (PDI) ranged from 0.110 to 0.261, indicating that the liposomes were homogeneously dispersed.

Both DPPC:Pluronic F-127 and DMPC:Pluronic F-127 systems stored at 4 °C showed a tendency to collapse over time.

**Figure 3:**
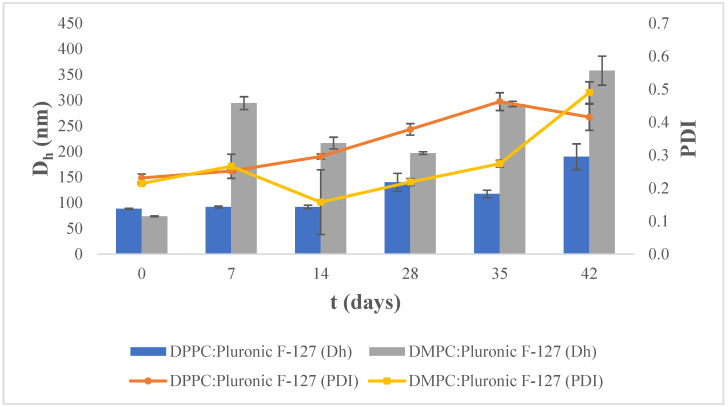
Stability assessment of the size (D_h_, nm) and polydispersity index (PDI) of DPPC:Pluronic F-127 and DMPC:Pluronic F-127 7:0.17 molar ratio over time at 25 °C. For D_h_ of all nanosystems, *p*-value < 0.0001. For PDI of all nanosystems, *p*-value < 0.0001.

As shown in Figure 3, at 25 °C, the mean hydrodynamic diameter of the DPPC:Pluronic F-127 system shows a continuous increase. The polydispersity index increased from the day of preparation (t = 0 d) continuously until the day t = 35 d, after which shows a decrease. Conversely, on day t = 42 d, when the hydrodynamic diameter takes its maximum value, the value of the polydispersity index decreased.

The mean hydrodynamic diameter (D_h_) of DMPC:Pluronic F-127 liposomes stored at 25 °C shows significant fluctuations over a period of 42 days. The polydispersity index (PDI) appears to increase over time, similar to the DPPC:Pluronic F-127 nanosystem stored at the same temperature (25 °C). This increase in the PDI value indicates the existence of various populations in the colloidal dispersion and the gradual collapse of the system.

We observed that the maximum values of the hydrodynamic diameter of the nanosystems at 4 °C are generally smaller than the maximum values of the hydrodynamic diameter at 25 °C.

The above systems at both storage temperatures show a tendency to collapse over time. This result could be attributed to the presence of the polymer at a concentration of 2.4 mol %. Johnson et al. reported that Pluronic F-127 at approximately 2 mol % can increase liposome size due to the formation of bilayer disks [19]. The smaller mean hydrodynamic diameter of DMPC:Pluronic F-127 liposomes compared to DPPC:Pluronic F-127 on the day of preparation is in agreement with the literature as it is argued that the shorter alkyl chain of the lipid leads to smaller liposome size (Table 1). According to a study conducted by Calori et al., in lipid–surfactant interactions, factors such as the main phase transition temperature and the molecular weight of the lipid play a secondary role in influencing the hydrodynamic diameter, while hydrophobicity of the polymer plays a dominant role [34,35,36]. Thus, the physicochemical profiles of DPPC:Pluronic F-127 and DMPC:Pluronic F-127 systems are consistent with these findings.

Pluronic F-127 is a thermoresponsive triblock copolymer. Micelles are formed at concentrations of Pluronic F-127 above the critical micelle concentration (CMC) or above the critical micelle temperature (CMT) at a specified concentration [37]. At 25 °C, the CMC is 0.555 mM, which is considerably lower than the surfactant concentration used; therefore, micelles could be formed [34]. However, as in our study, micelle formation can be limited when the polymer is added during liposome preparation, as it is more likely to be incorporated between the lipids of the bilayer as liposomes are formed [19]. Specifically, at 4 °C, fewer Pluronic F-127 molecules could interact with the membrane, some being translamellar, and others interacting with only one side of the lipid membrane [37]. In conclusion, we suggest that, at 25 °C, the interaction between the surfactant and lipid is greater, leading to higher hydrodynamic diameters. The smaller liposomal sizes observed in both systems at 4 °C could be attributed to the lower presence of Pluronic F-127 molecules in the lipid bilayer [34].

### 3.2. Physicochemical Characterization of the Quercetin-Loaded Nanoparticles

Regarding quercetin-loaded liposomes, the mean hydrodynamic diameter (D_h_) and the polydispersity index (PDI) were also studied at two different temperatures (4 °C and 25 °C), over a period of 42 days (Table 2). The stability of these systems was assessed as well.

**Figure 4:**
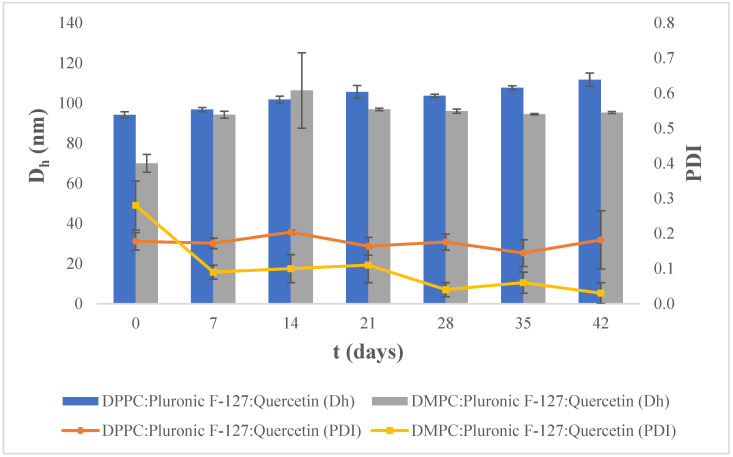
Stability assessment of the size (D_h_, nm) and polydispersity index (PDI) of DPPC:Pluronic F-127:quercetin and DMPC:Pluronic F-127:quercetin 7:0.17:0.5 molar ratio over time at 4 °C. For D_h_ of DPPC:Plur:Que, *p*-value > 0.05 and DMPC:Plur:Que *p*-value < 0.0001. For PDI of DPPC:Plur:Que, *p*-value > 0.05, and for DMPC:Plur:Que, *p*-value < 0.01.

As shown in Figure 4, at 4 °C, the DPPC:Pluronic F-127:quercetin liposomal nanosystem exhibits a small hydrodynamic diameter that remains stable over the 42-day period, with small day-to-day variations not exceeding 5 nm. The liposomal nanosystem composed of DMPC:Pluronic F-127:quercetin also appears to remain physicochemically stable over the study period, even though it exhibits greater fluctuations in values of the hydrodynamic diameter compared to DPPC liposomes. The size of DMPC liposomes initially increases from 70 nm to 94 nm, where it remains relatively stable with small deviations (except on day 14). Regarding the PDI, we observed that both systems show low PDI values, indicating the presence of monodispersion. However, the DPPC:Pluronic F-127:quercetin liposomal nanosystem is characterized by greater stability, as the physicochemical properties of the liposomes exhibit much smaller fluctuations.

**Figure 5:**
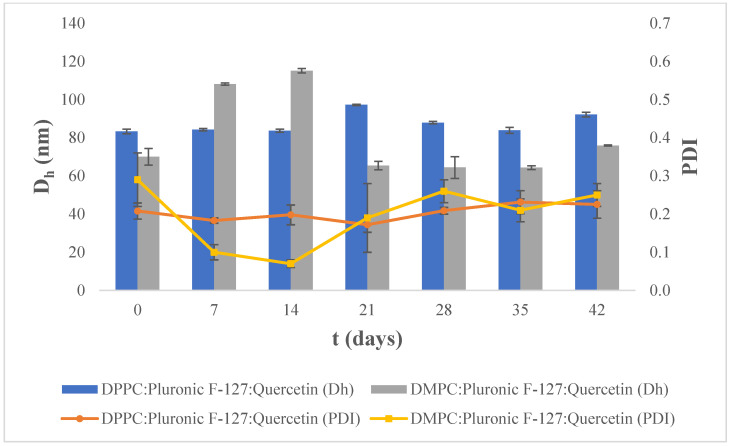
Stability assessment of the size (D_h_, nm) and polydispersity index (PDI) of DPPC:Pluronic F-127:quercetin and DMPC:Pluronic F-127:quercetin 7:0.17:0.5 molar ratio over time at 25 °C. For D_h_ of DPPC:Plur:Que, *p*-value > 0.05, and for DMPC:Plur:Que, *p*-value < 0.0001. For PDI of DPPC:Plur:Que, *p*-value < 0.01, and for DMPC:Plur:Que, *p*-value < 0.0001.

The systems were also stored at 25 °C, and the results from the stability study are shown in Figure 5. Regarding hydrodynamic diameter, we observe that the DPPC:Pluronic F-127:quercetin liposomal nanosystem remains stable over the study period, with small day-to-day differences. The DMPC:Pluronic F-127:quercetin liposomal nanosystem exhibits small hydrodynamic diameter values, which remain relatively stable over the 42-day period, although they show a large increase up to day 14. Regarding the PDI, we observe that PDI values of the DPPC:Pluronic F-127:quercetin liposomal nanosystem are low (lower than 0.250) and remain stable over time, indicating homogeneity in liposome size. The liposomal nanosystem composed of DMPC:Pluronic F-127:quercetin also appears to have low PDI values (lower than 0.300), indicating the presence of monodispersion.

We conclude that the prepared liposomal nanosystems do not exhibit significant differences in their physicochemical characteristics depending on their storage temperature. The incorporation of quercetin into the liposome structure leads to physicochemically stable liposomal nanosystems regardless of the lipid or the storage temperature, maintaining low values of the hydrodynamic diameter and the PDI over time.

According to Table 2, the liposomal nanosystems maintain their hydrodynamic diameter and PDI values over the 42-day period, a fact attributed to the components of the liposomes. Regarding the physical stability of the liposomal nanosystems, Pluronic F-127 was expected to positively affect their physical stability through the steric stabilization it provides due to its position in the lipid bilayer [20]. The hydrophobic part of Pluronic F-127 (PPO) is incorporated within the aliphatic chains of the lipid bilayer and its hydrophilic parts (PEOs) can either both extend towards the same side of the aqueous environment, or one can project towards the external and the other towards the internal aqueous environment [19,38]. Thus, the hydrophilic blocks of Pluronic F-127 extending into the external aqueous environment of the liposomes prevent their fusion both due to steric and enthalpic stabilization. When liposomes approach each other, the water molecules hydrating the hydrophilic blocks of the polymer are released, resulting in an increase in enthalpy, which leads to the repulsion of the particles (enthalpic stabilization) [13]. However, based on the physicochemical characteristics of the non-loaded liposomes, it appears that the steric and enthalpic stabilization provided by Pluronic F-127 is not sufficient to fully maintain the physical stability of these systems during the study period. Therefore, we conclude that quercetin significantly contributes to the physical stability of the liposomal nanosystems.

Quercetin is a molecule that exhibits a flat structure and is characterized by the presence of both hydrophobic and hydrophilic groups [39]. Quercetin possesses five hydroxyl groups, the protons of which are exchangeable [40]. As the pH of the dispersion medium increases, the hydroxyl groups of quercetin deprotonate, enhancing its hydrophilic character [39,40]. According to the literature [41], at pH = 5.5, which is the pH of the dispersion medium that is prevalent in our study, a negatively charged group is present on the quercetin molecule. This finding is also supported by the slightly more negative ζ-potential value obtained for the quercetin-loaded nanoparticles (Table 3). Based on the physicochemical characteristics observed during the stability study of the systems, it appears that quercetin, due to its negatively charged group, interacts with the lipid bilayers of the liposomes in the manner described for physiological pH conditions, where the deprotonated forms of quercetin are predominant, leading to the increased interaction of quercetin with the polar heads of phospholipids [39,42]. The polar heads of phospholipids, and specifically the C-O-P-O-C segment, interact with quercetin either through electrostatic bonds with the negatively charged groups or through hydrogen bonds with the hydroxyl groups that are not deprotonated at that particular pH value [40,42]. Therefore, quercetin positions itself towards the surface of the lipid bilayer. This extensive interaction with the polar heads of the phospholipids affects the distance between them, increasing the space occupied by each lipid, which results in a reduction in hydrophobic van der Waals interactions between the lipid chains and a decrease in their packing density, ultimately leading to increased fluidity of the lipid bilayer [39,42,43].

This increase in the fluidity of the lipid bilayers of the liposomes caused by quercetin positively affects the physical stability of the liposomal nanosystems. As mentioned, the hydrophilic clusters of Pluronic F-127 that extend towards the liposome external environment provide steric and enthalpic stabilization. However, they can also lead to stability issues. More specifically, the increase in the amount of Pluronic F-127 in the lipid bilayer leads to the development of lateral tension between its hydrophilic parts, which results in their transition from a relaxed “mushroom” configuration to a “brush” configuration, where the chains are more extended [19]. The concentration at which this mushroom-to-brush transition occurs depends on the length of the polymer’s hydrophilic segment. For Pluronic F-127, it has been reported that, for liposomes whose surface does not differ significantly from a flat surface, i.e., for liposomes larger than 50 nm, this transition occurs at polymer concentrations greater than 2 mol % [19]. A further increase in polymer concentration leads to increased lateral tension due to repulsions developing between the hydrophilic segments of the polymers. The reduction in lateral tension is achieved by increasing the curvature of the liposome structure [19]. Therefore, we conclude that, in our case, where the concentration of Pluronic F-127 is 2.4 mol %, it is more likely that the polymer has transitioned to the “brush” configuration. Quercetin, through its incorporation into the lipid bilayer, increases the fluidity, which allows the lipid bilayer to increase its curvature, leading to a reduction in lateral tension [19,42,43]. The increased stability of these systems, compared to non-loaded counterparts, could be attributed to the ability of liposomes to form higher-curvature structures without disruption, combined with the enthalpic stabilization provided by Pluronic F-127.

### 3.3. Entrapment Efficiency of Quercetin and the Effect on ζ-Potential

In Table 3 we observe that the DPPC:Pluronic F-127 and DMPC:Pluronic F-127 systems have a slight negative charge close to zero. This finding is due to the zwitterionic nature of lipids and the typically zero charge of Pluronic F-127 when the latter is in an aqueous environment. In contrast, the DPPC:Pluronic F-127:quercetin and DMPC:Pluronic F-127:quercetin 7:0.17:0.5 systems have a stronger negative charge, and, thus, electrostatic repulsions occur between the liposomes. As we already mentioned, at pH = 5.5, which corresponds to the pH of the dispersion medium in our study, a negatively charged group is present on the quercetin molecule. For this reason, the stronger negative charge of quercetin systems is justified. Regarding the entrapment efficiency, we found that the DMPC:Pluronic F-127:quercetin system entrapped 92% quercetin, in contrast to the DPPC:Pluronic F-127:quercetin system that had a lower percentage. This result could be attributed to the more fluid nature of DMPC:Plur, as it is evident from DSC studies presented at a later section.

### 3.4. The Effect of Serum Proteins on the Physicochemical Behavior of Developed Nanosystems

**Figure 6:**
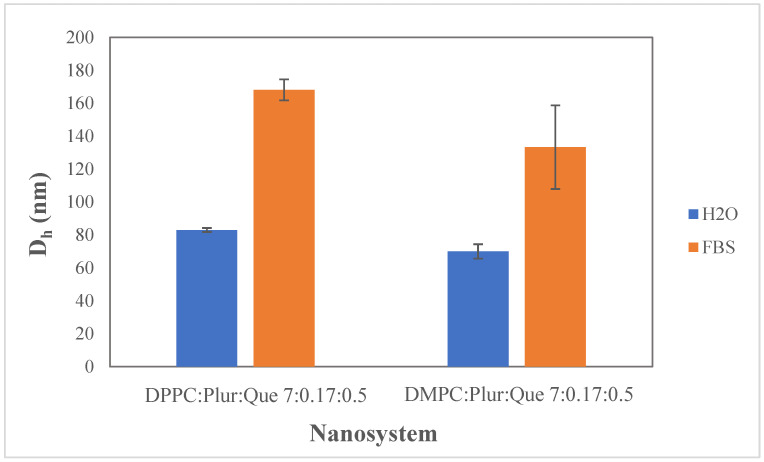
Hydrodynamic diameter (D_h_, nm) of DPPC:Pluronic F-127:quercetin 7:0.17:0.5, DΜPC:Pluronic F-127:quercetin 7:0.17:0.5 in aqueous and biological medium (FBS). For D_h_ of DPPC:Plur:Que, *p*-value < 0.01 and DMPC:Plur:Que *p*-value > 0.05.

The physicochemical properties of the quercetin-loaded liposomal nanosystems were characterized after incubation in FBS on the day of their preparation (t = 0 day) (Figure 6).

We observe that the hydrodynamic diameter values increase for both systems after incubation in FBS. Specifically, a larger increase in the hydrodynamic diameter is observed for the DPPC:Pluronic F-127:quercetin compared to the DMPC:Pluronic F-127:quercetin liposomal nanosystem. However, despite the presence of proteins, the size remains below 170 nm, demonstrating minimal protein binding to the surface of the liposomes, due to the incorporation of Pluronic F-127 in the structure of liposomes. According to the literature [18], Pluronics, which are copolymers based on PEG, recognized as the gold standard for long circulating nanoparticles, consist of two hydrophilic (PEO) blocks that inhibit protein adhesion. Generally, the presence of polymers on the surface of liposomes provides a form of steric stabilization, as the flexible chains of polymers cover the surface of the liposomes, preventing the interaction with components of the biological environment [44]. This behavior decreases the immunogenicity of the liposomes, reduces clearance by the reticuloendothelial system (RES), and consequently extends the duration of blood circulation, demonstrating a ‘stealth effect’ [18].

**Figure 7:**
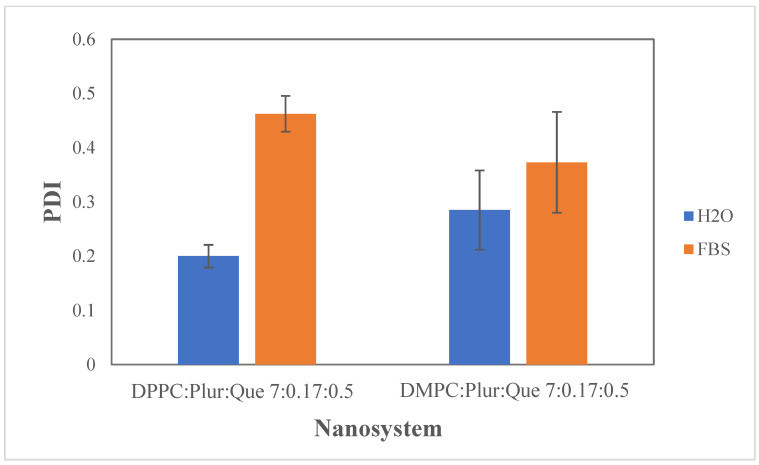
The polydispersity index (PDI) of DPPC:Pluronic F-127:quercetin 7:0.17:0.5, DΜPC:Pluronic F-127:quercetin 7:0.17:0.5 in aqueous and biological medium (FBS). For PDI of DPPC:Plur:Que, *p*-value < 0.01, and for DMPC:Plur:Que, *p*-value > 0.05.

As shown in Figure 7, after incubation in the FBS, an increase in the polydispersity index (PDI) was observed in both systems with entrapped quercetin. More specifically, the PDI value in the DPPC:Pluronic F-127:quercetin 7:0.17:0.5 system doubled, indicating the presence of multiple-size populations in the colloidal dispersion. Regarding the DMPC:Pluronic F-127:quercetin 7:0.17:0.5 system, the PDI increased from 0.285 to 0.373, indicating greater colloidal dispersion heterogeneity.

### 3.5. Thermotropic Evaluation of the Developed Lipid Bilayers

Differential scanning calorimetry (DSC) was used to obtain the thermodynamic parameters and to study the thermotropic behavior of the lipid bilayers prepared in this study. The results obtained through thermal analysis further explain and verify the physicochemical behavior of the lipid nanosystems, which has been extensively analyzed in the corresponding section. The results demonstrated that the phenomena were repeatable, as the same behavior was observed after one heating–cooling cycle, followed by the additional heating of all the examined lipid bilayers.

**Figure 8:**
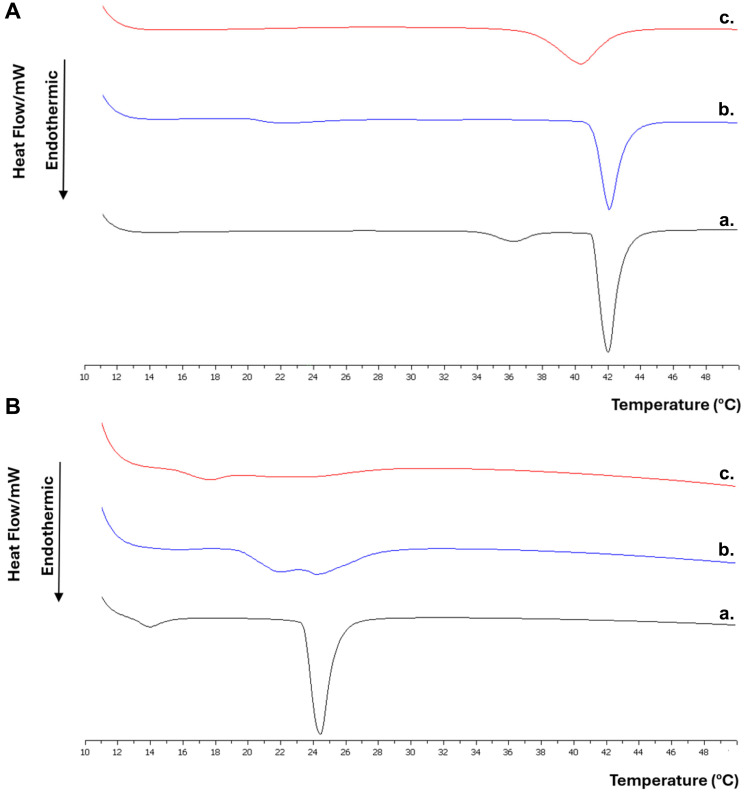
DSC heating scans of (**A**). DPPC (**a**), DPPC:Pluronic F-127 7:0.17 (**b**), DPPC:Pluronic F-127:quercetin 7:0.17:0.5 (**c**), and (**B**). DMPC (**a**), DMPC:Pluronic F-127 7:0.17 (**b**), and DMPC:Pluronic F-127:quercetin 7:0.17:0.5 (**c**) in the temperature range from 10 °C to 50 °C (5 °C/min scanning rate).

As shown in Figure 8, the pure DPPC bilayers exhibit two endothermic phase transitions with temperature increasing from 10 °C to 50 °C. The first transition observed is a broad, low-enthalpy pretransition from the gel phase (Lβ′) to the rippled phase (Pβ′), which occurs at 36.1 °C and is attributed to the mobility of the DPPC polar heads [45]. The mobility of the aliphatic chains of DPPC leads to the main transition from the rippled phase (Pβ′) to the liquid crystal phase (Lα), which appears as a sharp, high-enthalpy peak with a melting point at 41.7 °C [45].

**Figure 9:**
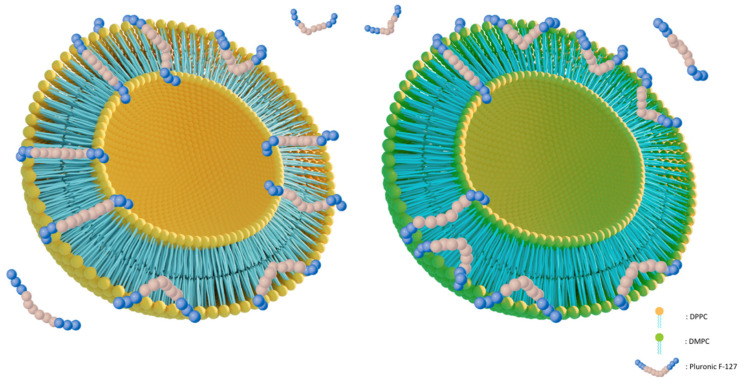
Schematic illustration of the different ways that Pluronic F-127 incorporates into DPPC and DMPC lipid bilayers.

Regarding the DPPC:Pluronic F-127 lipid bilayers, we observe that the incorporation of the polymer into the liposome structure did not significantly affect the thermotropic behavior of the system. The pretransition peak of DPPC becomes broader, and the temperature at which it appears decreases, as expected when a second component is added to the lipid bilayer, but it does not disappear. The above indicate that Pluronic F-127 interacts with the polar heads of the phospholipids in the lipid bilayer, but not to a large extent. This finding can be explained by the manner in which Pluronic F-127 presumably integrates into the liposome’s lipid bilayer (Figure 9). Specifically, the addition of Pluronic F-127 during the liposome preparation leads to most of it embedding along the lipid bilayer, resulting in its hydrophilic clusters projecting one towards the external and the other towards the internal aqueous environment. A much smaller proportion of it embeds in the bilayer with both hydrophilic clusters projecting towards the same side of the aqueous environment [19]. This parallel alignment of Pluronic F-127 with the lipids in the bilayer results in minimal interaction with the polar heads of the phospholipids and the fatty chains, as evidenced by the slight impact on the main phase transition temperature of DPPC. Additionally, we observe that the width of the DPPC main transition peak increases, but the ΔT_1/2_ value remains small, indicating good cooperativity between DPPC and Pluronic F-127. This finding suggests that Pluronic F-127 is uniformly distributed within the lipid bilayer without forming regions with different surfactant distributions (nanodomains). Finally, we observe a decrease in the enthalpy change for the two-phase transitions, indicating that the transition from one phase to another occurs more easily.

Regarding the DPPC:Pluronic F-127:quercetin lipid bilayers, we observe that the addition of a third component, quercetin, significantly affects the thermotropic behavior of the system compared to the addition of only the polymer. In this case, the pretransition peak disappears, demonstrating that quercetin interacts with the polar heads of phospholipids. Moreover, a decrease in the main phase transition temperature (T_m_) of DPPC is observed. This decrease is expected since quercetin, due to its position, interacts not only with the polar heads but also with the hydrophobic tails of the phospholipids through its hydrophobic rings [39]. According to the literature [39], the incorporation of quercetin into lipid bilayers of phospholipids with saturated fatty acid chains leads to a lowering of their melting point, resulting in systems with increased fluidity. In this particular case, the reduction in the main phase transition temperature is not significant, resulting in a system that gains greater fluidity without fully liquefying, which would otherwise lead to its collapse. Regarding enthalpy, it decreases, but not significantly, especially considering the addition of a third component to the lipid bilayer. As a result, the system requires less energy to transition from the rippled phase to the liquid crystalline state, without becoming much more sensitive to external factors. These findings support the observations from the stability study of the systems, indicating that quercetin imparts fluidity to the liposomes [39,42,43] without destroying them, leading to further stabilization of these systems. Finally, it is observed that the breadth of the main phase transition peak increases more compared to the increase observed with the addition of Pluronic F-127. This finding implies the formation of more distinct regions with different surfactant distributions.

The lipid bilayers containing only DMPC also showed two endothermic phase transitions with increasing temperature, in the studied range. One was a broad low-enthalpy pretransition peak from gel phase (Lβ′) to rippled phase (Pβ′), which occurs at 13.8 °C, and the next was a sharp (main transition) from rippled phase (rippled phase, Pβ′) in a liquid crystal phase (liquid crystal phase, La) with a melting point of 24.1 °C (Table 4). In addition, the pretransition peak was affected and disappeared from all thermal analysis results of DMPC lipid bilayers due to the interaction of the hydrophilic head of the lipid with the hydrophilic part of Pluronic F-127 or the hydroxyls of quercetin.

In the thermogram of the DMPC:Pluronic F-127 system, we observe that the main phase transition peak splits into two smaller ones (Figure 8). The appearance of the double peak phenomenon is possibly due to the presence of two nanodomains with different structures (phase separation): a region rich in Pluronic F-127 and one poor. Pluronic F-127 is reported in other studies to be able to interact in two ways with the lipid membrane: with the partial partition of the PPO unit in the lipid acyl chain and with the total partition of the PPO unit by spanning the lipid bilayer [38,46]. To achieve the second conformation, in a PEO-PPO-PEO type surfactant, the PPO chain must be of appropriate length. In addition, the PPO unit shows a strong thermal dependence [47]. In the selected polymer, the PPO chain seems to have a sufficient length to be inserted between the hydrophobic chains of the lipids, with its hydrophilic clusters extending toward both the external and internal aqueous environment. Thus, the way the surfactant binds to the membrane and the effect of temperature on polymer incorporation are key parameters in understanding biomaterial interactions. Increasing temperature enhances lipid membrane fluidity, promoting greater polymer incorporation [48]. Therefore, the lipid phase plays a crucial role in the partition of Pluronic F-127 [48,49]. The lower main phase transition temperature of the DMPC:Pluronic F-127 system compared to pure DMPC indicates the incorporation of Pluronic F-127 into the membrane [22]. According to a study conducted by Chandaroy et al., increasing the temperature enhances the hydrophobicity of the PPO part of the polymer, resulting in its incorporation into the membrane. In fact, in the same study, it is argued that, due to the thermo-responsive property of the surfactant, if the temperature is lowered, the polymer dissociates from the membrane or micelles and dominates as a monomer in the colloidal dispersion [37]. Regarding the change in the phase transition enthalpy (ΔH) of the system, its values are smaller in the DMPC:Pluronic F-127 system than in pure DMPC due to the increased lipid–surfactant interactions compared to the lipid–lipid interactions because they are stronger and require more energy to “break” [50].

Differential scanning calorimetry (DSC) could be a tool that can provide insights into the physicochemical stability of systems composed of the current biomaterials. Regarding the different modes of interaction of Pluronic F-127 with the lipid membrane, we suggest that, in the DMPC:Pluronic F-127 system, V-type interactions prevail, where the polymer incorporates into the lipid membrane by entering and leaving the membrane on the same side of the vesicle (Figure 9), [51]. In this configuration, the distances between the lipids increase, leading to a higher hydrodynamic diameter. Thus, in liposomes stored at 25 °C, Pluronic F-127 interacted more extensively with the membrane, resulting in a higher liposome size. In contrast, at 4 °C, the surfactant dislodges from the membrane, leading to a lower liposome size and the prevalence of a Pluronic F-127-poor nanodomain.

In the DMPC:Pluronic F-127:quercetin system, a double peak appears again in the main phase transition peak. However, at this peak, phase separation is less distinct due to the poor cooperativity of the biomaterials, which increases the width (ΔT_1/2_) of the system. In addition, the main phase transition temperature of the system has been further reduced, apparently due to the addition of the third component (impurity). Quercetin is reported to increase membrane fluidity, resulting in greater polymer incorporation [52,53]. Also, the phase transition enthalpy change is higher compared to the DMPC:Pluronic F-127 system due to fewer lipid–lipid interactions, as previously explained [50]. Thus, we assume that the system is dominated by surfactant-rich nanodomains at both storage temperatures. This assumption is based on the observation that the system remains physicochemically stable, with similar mean hydrodynamic diameters, regardless of the storage temperature.

## 4. Conclusions

In conclusion, this study highlights the potential predictive value of DSC for preformulation liposomal stability studies. Among the systems studied, the DPPC:Pluronic F-127 system exhibited the most promising physicochemical and thermotropic characteristics for effective quercetin delivery. Future research should explore the system’s long-term stability and performance in various biological conditions to further assess its therapeutic potential.

## Figures and Tables

**Table 1:** The hydrodynamic diameter (D_h_) and polydispersity index (PDI) of DPPC:Pluronic F-127 (7:0.17) and DMPC:Pluronic F-127 (7:0.17) non-loaded nanosystems on the day of preparation and after 42 days of storage at 4 °C and 25 °C. The results are the mean ± standard deviation (n = 3).

Sample	Storage Temperature (°C)	D_h_ (nm)		PDI	
		Day 0	Day 42	Day 0	Day 42
DPPC:Plur 7:0.17	4	88.4 ± 1.2	144.0 ± 7.2	0.231 ± 0.012	0.532 ± 0.006
	25		190.0 ± 25.2		0.416 ± 0.040
DMPC:Plur 7:0.17	4	73.7 ± 0.9	247.2 ± 1.7	0.214 ± 0.002	0.245 ± 0.029
	25		357.8 ± 28.2		0.490 ± 0.033

**Table 2:** The hydrodynamic diameter (D_h_) and polydispersity index (PDI) of DPPC:Pluronic F-127:quercetin (7:0.17:0.5) and DMPC:Pluronic F-127:quercetin (7:0.17:0.5) nanosystems on the day of preparation and after 42 days of storage at 4 °C and 25 °C. The results are the mean ± standard deviation (n = 3).

Sample	Storage Temperature (°C)	D_h_ (nm)		PDI	
		Day 0	Day 42	Day 0	Day 42
DPPC:Pluronic:quercetin 7:0.17:0.5	4	83.3 ± 1.2	111.7 ± 3.3	0.208 ± 0.021	0.182 ± 0.083
	25		92.2 ± 1.2		0.225 ± 0.036
DMPC:Pluronic:quercetin 7:0.17:0.5	4	70.0 ± 4.4	95.3 ± 0.5	0.285 ± 0.073	0.029 ± 0.028
	25		75.9 ± 0.3	0.285 ± 0.073	0.251 ± 0.029

**Table 3:** The entrapment efficiency of the quercetin-loaded nanosystems and ζ-potential of all the prepared nanosystems on the day of preparation, t = 0 days. The results are the mean ± standard deviation (n = 3).

Sample	EE%	ζ-Potential (mV)
DPPC:Plur	-	−5.99 ± 1.01
DPPC:Plur:Que	79	−11.05 ± 1.23
DMPC:Plur	-	−3.46 ± 0.30
DMPC:Plur:Que	92	−11.19 ± 3.05

**Table 4:** Calorimetric profiles of DPPC, DPPC:Pluronic F-127, DPPC:Pluronic F-127:quercetin, DMPC, DMPC:Pluronic F-127, and DMPC:Pluronic F-127:quercetin hydrated in HPLC grade water. T_onset_: temperature at which the thermal event starts; T: temperature at which heat capacity (ΔC_p_) at constant pressure is maximum; ΔT_1/2_: half width at half peak height of the transition; ΔH: transition enthalpy normalized per gram of sample lipid bilayer.

Sample	Molar Ratio	Cycle	Τ_onset,m_ /°C	T_m_ /°C	ΔΤ_1/2m_ /°C	ΔH_m_ /Jg^−1^	Τ_onset,s_ /°C	T_s_ /°C	ΔΤ_1/2s_ /°C	ΔH_s_ /Jg^−1^
DPPC	-	Heat	41.0	41.7	1.15	−39.0	34.3	36.1	2.03	-5.3
DPPC:Plur	7:0.17	Heat	40.9	41.8	1.21	−31.3	20.0	22.0	3.84	-3.8
DMPC:Plur:Que	7:0.17:0.5	Heat	37.3	40.1	2.82	−28.5	-	-	-	-
DMPC	-	Heat	23.3	24.1	1.22	−33.4	11.8	13.8	1.94	-5.7
DMPC:Plur	7:0.17	Heat	20.2	24.0	5.85	−27.7	-	-	-	-
DMPC:Plur:Que	7:0.17:0.5	Heat	14.8	17.5	9.46	−20.9	-	-	-	-

## Data Availability

The original contributions presented in the study are included in the article, further inquiries can be directed to the corresponding author.
